# Supplementary material for: Preparations of Dutch emergency departments for the COVID-19 pandemic: A questionnaire-based study
Source: PLoS One. 2021 Sep 10;16(9):e0256982. doi: 10.1371/journal.pone.0256982 (PMC8432867; doi:10.1371/journal.pone.0256982)
Supplement: S1 File — (DOCX) [file pone.0256982.s001.docx]

## S1 File - Questionnaire (English)

1. What is the annual ED attendance?
   - <20.000 patients
   - 20.000-25.000 patients
   - 25.000-30.000 patients
   - 30.000-35.000 patients
   - 35.000-40.000 patients
   - >40.000 patients
2. Is the ED staffed by EPs?
   - Yes
   - No
3. How many treatment places does the ED have?
4. Have there been preparations with regards to the ED in anticipation of COVID-19 patients?
   - Yes
   - No
5. On what date were these preparations implemented? .. - ..-2020
6. Was the number of ED treatment places expanded because of the COVID-19 pandemic?
   - Yes, it was expanded to … ED treatment places (including treatment places for delivering standard emergency care and COVID-19 emergency care).
   - No. Could you elaborate why the number of ED treatment places was not expanded: …
7. Were arrangements made to relieve the ED?
   - Yes. Could you elaborate on these arrangements? …
   - No
8. Was the flow of COVID-19 patients needing emergency care segregated from the flow of standard emergency department patients?
   - No
   - Yes
     1. Where did the emergency care for COVID-19 patients take place?
        - Original ED only
        - Original ED and other location: Which was/were …
        - Only other location: Which was …
     2. Where did the emergency care for COVID-19 patients take place?
        - Original ED only
        - Original ED and the following other location: Which was …
        - Only other location: Which was …
9. If COVID-19 care was segregated, screening for COVID-19 before ED entry was performed by:
   1. Symptom-based screening list:
      - Yes
      - No
   2. Chest X-ray
      - Yes
      - No
   3. Chest CT
      - - Yes
        - No
10. Was ED personnel capacity increased because of the COVID-19 pandemic?
    - No
    - Yes
      1. Additional ED nurses were deployed
         - Yes
         - No
      2. Additional non-ED nurses were deployed
         - Yes
         - No
11. Were the following specialties involved in the COVID-19 care in the ED?
    1. Anaesthesiology
       - Yes
       - No
    2. Cardiology
       - Yes
       - No
    3. Dermatology
       - Yes
       - No
    4. Emergency medicine
       - Yes
       - No
    5. Gastro-enterology
       - Yes
       - No
    6. Geriatrics
       - Yes
       - No
    7. Gynaecology
       - Yes
       - No
    8. Internal medicine
       - Yes
       - No
    9. Neurology
       - Yes
       - No
    10. Orthopaedics
        - Yes
        - No
    11. Otolaryngology/ENT
        - Yes
        - No
    12. Paediatrics
        - Yes
        - No
    13. Plastic surgery
        - Yes
        - No
    14. Primary care
        - Yes
        - No
    15. Pulmonology
        - Yes
        - No
    16. Rheumatology
        - Yes
        - No
    17. Surgery
        - Yes
        - No
    18. Urology
        - Yes
        - No
    19. Other
        - Yes, could you specify? …
12. Were the following disciplines involved in the COVID-19 care in the ED?
    1. Anesthetic nurses
       - Yes
       - No
    2. Medical interns
       - Yes
       - No
    3. Doctors’ assistants
       - Yes
       - No
    4. *Orthopedic practitioners*
       - Yes
       - No
    5. Surgery assistants
       - Yes
       - No
    6. Physician assistants
       - Yes
       - No
    7. Other. Coud you specify? …
13. Did the hospital organisation have multiple ED locations?
    - No
    - Yes
14. Did EPs have a role in COVID-19 related emergency care?
    - No
    - Yes
      1. EPs were directly involved in the assessment and treatment of COVID-19 patients
         - Yes
         - No
      2. EPs had a coordinating role regarding COVID-19 ED care.
         - Yes
         - No
      3. EPs were involved in triage or segregation of COVID-19 suspected patients
         - Yes
         - No
15. Did EPs have a formal role in the hospital’s crisis organization?
    - No
    - Yes
      1. EPs were member of the strategic crisis team
         - Yes
         - No
      2. EPs were member of an operational crisis team
         - Yes
         - No
16. *In the spring of 2020, during the peak of the pandemic in the Netherlands, was there a moment (or moments) when the capacity of the ED was insufficient?*?
    - Yes, occasionally
    - Yes, multiple times a week
    - Yes, daily
    - Yes, multiple times a day
    - No
17. Do you expect that measures that were implemented in the ED due to the COVID-19 pandemic will be instated permanently?
    - Yes, could you elaborate on these measures? …
    - No
