# Supplementary material for: Preparations of Dutch emergency departments for the COVID-19 pandemic: A questionnaire-based study
Source: PLoS One. 2021 Sep 10;16(9):e0256982. doi: 10.1371/journal.pone.0256982 (PMC8432867; doi:10.1371/journal.pone.0256982)
Supplement: S1 Table — (DOCX) [file pone.0256982.s003.docx]

## S1 Table – Alternative locations of ED care

| Location for COVID-19 ED care | EDs^*^ |
| --- | --- |
| Tent or decontamination unit adjacent to the ED | 6 (9.1%) |
| COVID outpatient department (in case of stable vitals) | 4 (6.1%) |
| Other hospital** | 4 (6.1%) |
| Fast lane cardiology clinic | 2 (3.0%) |
| Fast lane respiratory clinic | 2 (3.0%) |
| Pulmonologists and intensivists did consultations of patients at General Practice Cooperative adjacent to ED and decided to either admit to ward or send patient home | 2 (3.0%) |
| Space in specialised facility (such as minor surgery and endoscopy unit) | 1 (1.5%) |
| COVID screening department | 1 (1.5%) |

* numbers are presented as n (%)

**Hospital was designated as non-COVID-19 location

| Location for non-COVID ED care | EDs^*^ |
| --- | --- |
| Outpatient department (in case of stable vitals)** | 28 (42.4%) |
| Department adjacent to ED | 8 (12.1%) |
| Fast lane cardiology clinic | 6 (9.1%) |
| Other non-COVID ED location was set up within hospital | 5 (7.6%) |
| Acute admission department | 2 (3.0%) |
| Other hospital*** | 2 (3.0%) |
| Tent | 2 (3.0%) |
| ED care for specific patient groups |  |
| Children in paediatric ward or outpatient department | 12 (18.2%) |
| Thrombolysis eligible stroke patients in stroke unit | 3 (4.5%) |

* numbers are presented as n (%)

****** These patients included children and for example patients suffering minor traumatic injuries, oncologic disease and deep venous thrombosis.

***Hospital was designated as COVID-19 location
