# Supplementary material for: Preparations of Dutch emergency departments for the COVID-19 pandemic: A questionnaire-based study
Source: PLoS One. 2021 Sep 10;16(9):e0256982. doi: 10.1371/journal.pone.0256982 (PMC8432867; doi:10.1371/journal.pone.0256982)
Supplement: S2 Table. Measures intended as permanent — (DOCX) [file pone.0256982.s004.docx]

## S2 Table – Measures implemented due to COVID-19 that are intended as permanent.

| Measure | Hospital organizations* |
| --- | --- |
| Improved infection prevention | 14 (24.1%) |
| Improved interdisciplinary collaboration | 9 (15.5%) |
| Permanent adjustments to segregate possibly contagious patient categories | 7 (12.1%) |
| Permanent redirection of low-urgent patient categories | 7 (12.1%) |
| expansion of staff | 5 (8.6%) |
| Dynamic ED capacity model with adjacent hospital department (i.e. acute medical unit) | 3 (5.2%) |
| E-health/telemedicine | 1 (1.7%) |
| Permanent expansion of capacity | 3 (5.2%) |
| Improved throughput | 5 (8.6%) |
| More prominent role of emergency department | 2 (3.4%) |
| Extra diagnostic modalities | 1 (1.7%) |
| Improved transmural collaboration | 2 (3.4%) |
| Improved security (of staff and facility) | 1 (1.7%) |
| National coordination of clinical capacity | 1 (1.7%) |

* Data are presented as n (%)

Abbreviations: ED – emergency department
