# Supplementary material for: Preparations of Dutch emergency departments for the COVID-19 pandemic: A questionnaire-based study
Source: PLoS One. 2021 Sep 10;16(9):e0256982. doi: 10.1371/journal.pone.0256982 (PMC8432867; doi:10.1371/journal.pone.0256982)
Supplement: Attachment — Submitted filename: Response to Reviewers.docx [file pone.0256982.s008.docx]

Journal requirements:

1. Reference list:

*The reference list was reviewed and minor adjustments to the style were made to meet PLOS requirements. None of the references were removed or retracted.*

Response to reviewers’ comments:

*We would like to thank the reviewers for their thoughtful comments and efforts towards improving our manuscript. The minor issues were adjusted according to their suggestions.*

Reviewer #1: The responses are clear and I thank the authors for incorporating many of my suggestions. Two very minor issues:

- "Plaster technicians" is still in Table 3.

*‘Plaster technician’ was alterered to ’Orthopedic practitioner’*

- ln 178. The definition of crowding should be mentioned in the methods, not here.

*The sentence was moved to lines 107-108 of the methods.*
